# Diagnostic Balance Tests for Assessing Risk of Falls and Distinguishing Older Adult Fallers and Non-Fallers: A Systematic Review with Meta-Analysis

**DOI:** 10.3390/diagnostics10090667

**Published:** 2020-09-03

**Authors:** Žiga Kozinc, Stefan Löfler, Christian Hofer, Ugo Carraro, Nejc Šarabon

**Affiliations:** 1 Faculty of Health Sciences, University of Primorska, Polje 42, SI-6310 Izola, Slovenia; ziga.kozinc@fvz.upr.si; 2 Andrej Marušič Institute, University of Primorska, Muzejski trg 2, SI-6000 Koper, Slovenia; 3 Physiko- & Rheumatherapie, Institute for Physical Medicine and Rehabilitation, 3100 St. Pölten, Austria; stefan.loefler@rehabilitationresearch.eu; 4 Centre of Active Ageing—Competence Centre for Health, Prevention and Active Ageing, 3100 St. Pölten, Austria; 5 Ludwig Boltzmann Institute for Rehabilitation Research, Neugebäudeplatz 1, 3100 St. Pölten, Austria; christian.hofer@rehabilitationresearch.eu; 6 Department of Biomedical Sciences, University of Padova, Via Ugo Bassi, 58/B, 35131 Padova, Italy; ugo.carraro@unipd.it; 7 Interdepartmental Research Center of Myology, University of Padova, Via Ugo Bassi, 58/B, 35131 Padova, Italy; 8 A&C M-C Foundation for Translational Myology, Padova, Galleria Duomo 5, 35141 Padova, Italy; 9 InnoRenew CoE, Livade 6, SI6310 Izola, Slovenia; 10 Laboratory for Motor Control and Motor Behavior, S2P, Science to Practice, Ltd., Tehnološki park 19, SI-1000 Ljubljana, Slovenia

**Keywords:** older adults, falls, fall history, body sway, functional reach, single-leg test, Romberg test

## Abstract

Falls are a major cause of injury and morbidity in older adults. To reduce the incidence of falls, a systematic assessment of the risk of falling is of paramount importance. The purpose of this systematic review was to provide a comprehensive comparison of the diagnostic balance tests used to predict falls and for distinguishing older adults with and without a history of falls. We conducted a systematic review of the studies in which instrumented (force plate body sway assessment) or other non-instrumented balance tests were used. We analyzed the data from 19 prospective and 48 retrospective/case-control studies. Among the non-instrumented tests, the single-leg stance test appears to be the most promising for discrimination between fallers and non-fallers. In terms of body sway measures, the center-of-pressure area was most consistently associated with falls. No evidence was found for increased benefit of the body sway test when cognitive tasks were added, or the vision was eliminated. While our analyses are limited due to the unbalanced representation of different test and outcome measures across studies, we can recommend the single-leg test for the assessment of the risk of falling, and the measurements of body sway for a more comprehensive assessment.

## 1. Introduction

Falls are a major cause of injury, functional disability and morbidity in older adults [1,2]. In addition, the annual medical costs attributable to falls have been estimated at up to USD 50 billion in the United States alone [3]. A considerable amount of research has been devoted to exploring the effectiveness of various interventions to reduce the incidence and severity of falls [2,4]. The best strategy for reducing the risk of falls appears to be a multi-component exercise program, that includes strength, endurance and balance training [5]. In addition, systematic assessment of fall risk is crucial to reduce the incidence of falls in the older adult population [6]. Several instrumented and non-instrumented tests, as well as questionnaires for assessment of fall risk have been proposed and evaluated [7,8,9,10,11,12,13]. Recently, it has been suggested that at least two screening tools should be used together to maximize the advantages of each for predicting the occurrence of falls [8]. In this paper, we will focus on the diagnostic tests of postural balance ability in terms of usefulness for predication of falls and discrimination between older adults with and without history of falls.

It is well documented that body sway characteristics assessed via center-of-pressure (CoP) analysis are sensitive to changes associated with ageing [14,15,16]. Moreover, CoP measures have also been associated with an increased risk of falling [17] and fall history [18,19] in older adults. Since the assessment of body sway is time-consuming and potentially expensive, various non-instrumented tests have been proposed as an alternative. One of the most common tools used to assess the function of older adults, the Timed-Up-and-Go test, has been reported to have high specificity (74%), but low sensitivity (31%) for prediction of falls [10]. Similarly, low sensitivity (30%) and high specificity (92%) for identifying falls risk among individuals with Parkinson’s disease has been reported for the functional reach test [20]. Thereby, using these tests is not optimal, as several individuals with high risk of falling are left out. Other functional tests, such as the Romberg test [21,22] and single-leg stance test [23] have also been explored and showed mixed but promising results.

Although many screening tools to predict falls have been investigated in the past, these tools have never been comprehensively compared before. The purpose of this paper was to conduct a systematic review of the studies that examined the usefulness of different balance tests for predicting falls or discriminating older adults with and without a history of falls. We considered instrumented body sway (i.e., CoP analysis) assessments, as well as non-instrumented balance tests. We hypothesized that both types of test will be capable of distinguishing older adults with and without a history of falls, while only the CoP parameters will prove useful for fall prediction.

## 2. Materials and Methods

### 2.1. Inclusion Criteria

Study inclusion criteria were structured according to PICOS tool [24]:

- Population (P): Male or female older adults. The criterion for inclusion was mean sample age ≥ 60.0 years. The study had to include either two groups (fallers and non-fallers) or a single cohort that was prospectively tracked for falls.
- Intervention (I): No allocated intervention.
- Comparisons (C): Fallers and non-fallers were compared.
- Outcomes (O): Any tests assessing balance ability, either instrumented (body sway assessment through CoP analysis using force plates or other technology) or non-instrumented (Romberg test, functional reach test, star excursion balance test, stance time in different postures, etc.)
- Study design (S): Prospective and retrospective cohort studies or case-control studies.

### 2.2. Search Strategy

Multiple databases of scientific literature (PubMed, Cochrane Central Register of Controlled Trials, PEDro and ScienceDirect) were searched in July 2020 with no restriction regarding the date of publication. For the databases that enable using Boolean search operators, we used the following combination of search key words: (fall risk OR history of falls OR non-fallers OR fall prediction OR fall prevention) AND (elderly OR older adults OR ageing) AND (center of pressure OR center-of-pressure OR body sway OR force plate OR force platform OR CoP OR center-of-pressure OR center of pressure OR Romberg test OR SEBT OR functional reach test OR single-leg stance OR Y-test OR Y test OR star excursion OR Romberg scale OR quiet stance OR tandem stance OR semi-tandem). When this was not possible, we used several reduced combinations of key words, including, but not limited to elderly (i.e., older adults) fall balance test, elderly fallers balance and balance test fall history. Additionally, reference lists of several systematic review articles on the topic of balance tests in older adults were carefully reviewed. Database search was performed independently by two authors (Z.K. and S.L.). Two reviewers (N.S. and S.L.) also screened the titles and the abstracts independently. Potentially relevant articles were read in full text, followed by additional reviewing for their eligibility.

### 2.3. Data Extraction

The data extraction was carried out independently by two authors (Z.K. and C.H.), and disagreements were resolved through consultation with other authors. The extracted data included: (a) the means and standard deviations for all eligible outcome measures for fallers and non-fallers; (b) other variables describing the characteristics of the balance tests, such as sensitivity, specificity, related risk/odds ratios and area under receiver operator characteristics (ROC) curve and (c) baseline demographics of participants (gender, age, body height, body mass, body mass index). Data were carefully entered into Microsoft Excel 2016 (Microsoft, Redmond, WA, USA). If the data were presented in a graphical rather than tabular form, Adobe Illustrator Software (version CS5, Adobe Inc., San Jose, CA, USA) was used to accurately determine the exact values of the data.

### 2.4. Data Analysis and Synthesis

The main data analyses were carried out in Review Manager (Version 5.3, Copenhagen: The Nordic Cochrane Centre, The Cochrane Collaboration, 2014). The meta-analysis was computed with an inverse variance method for continuous outcomes with a random-effects model. The pooled effect sizes were expressed as standardized mean difference (SMD) between fallers and non-fallers. Statistical heterogeneity among studies was determined by calculating the I-square (I^2^) statistics. According to Cochrane guidelines, the I^2^ statistics of 0% to 40% might not be important, 30% to 60% may represent moderate heterogeneity, 50% to 90% may represent substantial heterogeneity and 75% to 100% indicates considerable heterogeneity [25]. The threshold for statistical significance was set at *p*  ≤  0.05 for the main effect size and the subgroup difference tests. Data for risk measures as well as the sensitivity and the specificity were assessed qualitatively due to the smaller number of eligible studies and insufficient data for pooling mean effects.

## 3. Results

### 3.1. Summary of Search Results and Characteristics of Included Studies

**Figure 1:**
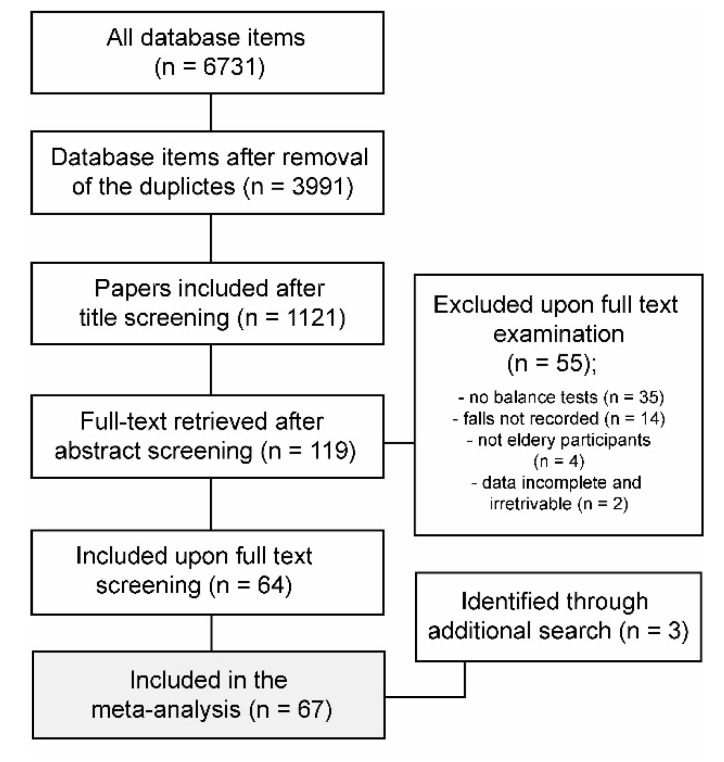
Flowchart of the article search protocol.

The results of the search steps are summarized in Figure 1. The search resulted in 67 studies in total. Three studies were retrieved through screening of previous systematic reviews. Altogether, there were 19 prospective studies [22,26,27,28,29,30,31,32,33,34,35,36,37,38,39,40,41,42,43] and 48 retrospective/case-control studies [18,19,21,23,44,45,46,47,48,49,50,51,52,53,54,55,56,57,58,59,60,61,62,63,64,65,66,67,68,69,70,71,72,73,74,75,76,77,78,79,80,81,82,83,84,85,86]. Body sway parameters were reported in 34 studies, while 38 studies included non-instrumented balance tests (5 studies included both). In prospective studies, the follow-ups lasted for 6 months (2 studies), 12 months (16 studies) or 24 months (1 study). In retrospective studies, the fall status was determined based on the occurrence of falls in the last 6 months (8 studies), 12 months (36 studies), 24 months (3 studies) or 60 months (1 study).

The protocol for assessment of body sway characteristics varied substantially between studies. Participants were barefoot in 18 studies and wore shoes in 2 studies, while 14 studies did not report the data regarding footwear. The participants had to place their hands on their hips (3 studies), behind their back (3 studies) or let the arms hang loose by their body (21 studies). The remaining 7 studies did not report the data regarding the position of the arms. The number of trials per condition was reported in 20 studies and ranged from 1 to 10 (3.45 ± 2.52 reps). The most common number of repetitions was 3 (7 studies). The duration of the trials was reported in 32 studies and ranged from 15 to 240 s (46.0 ± 38.62 s). The most common duration of the trials was 30 s (16 studies). Out of the 32 studies that included trials with eyes open, 11 reported that the participant had to focus on a particular point in the space (commonly a black dot on eye level). The age of the participants was reported in 65 studies. Across all studies, the mean age was 74.06 ± 5.75 years. The mean age for fallers and non-fallers was 74.51 ± 5.54 and 72.74 ± 5.06 years, respectively.

### 3.2. Discrimination of Fallers and Non-Fallers with Balance Tests

The ability to discriminate between older adult fallers and non-fallers was calculated for 48 body sway parameters and for 11 non-instrumented balance tests (Table 1). Note that the positive SMD indicates that the respective value is higher in faller groups and vice versa. Among the body sway parameters, it appears that the CoP sway area presented with the most consistently high SMD (0.30–0.67), with the exception of CoP sway area single-leg with eyes closed, which was higher in non-fallers (SMD = −0.31); however, this was derived only from one study. Overall, there was no clear pattern of direction specific (i.e., antero-posterior or medio-lateral) parameters to be better at discriminating fallers from non-fallers. Some of the parameters showed very high SMD (>0.80); however, these SMDs were typically based on a very small (1 3) number of studies. Among the parameters that appeared in at least 5 studies, the highest SMDs were shown for the CoP area in parallel stance with eyes open (SMD = 0.60 (0.20, 1.00)), medio-lateral CoP amplitude in parallel stance with eyes open (SMD = 0.35 (0.12, 0.58)) and eyes closed (SMD = 0.38 (0.12, 0.64)) and CoP path in parallel stance with eyes open (SMD = 0.34 (0.12, 0.56)).

### 3.3. Fall Risk Associated with Outcomes of Balance Tests

The risk of falling was reported for eight body sway parameters and for five non-instrumented balance tests (Table 2). Due to the small number of studies and heterogeneity regarding the risk type and cut-off values, the meta-analysis was not computed.

### 3.4. Sensitivity and Specificity of Balance Tests to Detect Fallers

The sensitivity and specificity to detect fallers was reported for two body sway parameters, and for two non-instrumented balance tests (Table 3). In general, the sensitivity was moderate to high for single-leg CoP velocity measures (0.70–78), low to high for functional reach test (0.47–0.75) and moderate for single-leg stance time (0.51–0.67). In contrast, the specificity was high only for single-leg stance time in one study (0.89) and low to moderate in other studies (0.43–0.67).

## 4. Discussion

The purpose of this systematic review was to provide an overview of the utility of different diagnostic balance tests for the older adults, in terms of assessing the risk of falling and distinguishing between individuals with and without a history of falls. Regarding the differences between fallers and non-fallers, 59 different outcome measures were evaluated. Among the CoP parameters, the CoP area appears to be the most consistently increased in fallers across studies. CoP amplitude and CoP path length were also promising in this view, while the differences between fallers and non-fallers were smaller for CoP velocity parameters and unclear for CoP frequency parameters. Functional reach test and single leg stance test were able to distinguish fallers from non-fallers, with the latter appearing superior. The other non-instrumented balance tests were included in a very limited number of studies. We found studies that reported good sensitivity of the CoP velocity (0.70–0.78) and functional reach (0.47–0.75) test and moderate sensitivity (0.51–0.67) of single leg stance test. On the other hand, the specificity of these tests was generally lower (0.43–0.67), with the exception of one study reporting high specificity for single-leg stance test (0.89). Different cut-off values for identification of individuals who are at high risk of falls have been found, and highly varying odds of prospective falls were reported across studies (measures of risk: 1.03–2.90 for CoP parameters, 0.5–8.67 for functional reach test and 0.38–15.22 for single-leg stance test).

Systematic assessment of the risk of falling is considered as a crucial step towards the reduction of the incidence of falls in older adult populations [6]. The first step in establishing an acknowledged screening tool is to test its reliability. Good or excellent reliability for the assessment in older adults has already been reported for the functional reach test [13,87,88], single-leg stance test [87,88] and Romberg test [88]. The single-leg stance test seems to be the most useful among the non-instrumented balance tests in terms of differentiating between fallers and non-fallers and for predicting falls (although the latter has not been statistically evaluated in this review). It has been suggested that the functional reach test is significantly influenced by the flexibility of trunk and voluntary neuromuscular control, and is, therefore, not the best measure of balance [78,89], while the single-leg stance test performance depends predominantly on medio-lateral balance control. On the other hand, it has been shown previously that certain screening instruments that are not considered balance tests (and are, therefore, not included in this review) are associated with falls in older people. In particular, the Timed-Up-and-Go test has been extensively investigated and recommended for fall risk assessment [10,90]. The utility of this test is usually explained by the fact that it reflects strength, balance and mobility [10].

The analysis of body sway during quiet stance has been extensively used in older adults, with high to excellent reliability consistently reported [15,91,92]. However, it remains unknown which task (i.e., stance) and which parameter is the best predictor of falls. In our analyses, the CoP area seemed to be most consistently associated with falls. It should be noted that some of the outcomes (notably parameters related to semi-tandem, tandem and single-leg stances) were reported much less frequently than others. Overall, it appears that measurements of CoP amplitude and area are more sensitive to falls than velocity measurements, which are in turn superior to frequency measurements. A previous review has indicated that examination of body sway with eyes closed may provide clearer insights into ageing-related changes [93]. However, we found no clear evidence that the assessment with eyes closed provides additional value for assessing the risk of falling. Moreover, there was no clear preference for direction specific (i.e., antero-posterior or medio-lateral) measures, except in view of the odds ratio pertaining to the fall risk based on the CoP amplitude (antero-posterior: 1.3–1.5; medio-lateral: 2.3–2.9). This is consistent with our results related to non-instrumented balance tests, as the single-leg stance test primarily stresses the medio-lateral balance, while the functional reach test, which was less sensitive to falls, primarily reflects the antero-posterior balance. 

It is well known that an additional cognitive task during quiet stance significantly increases body sway in older adults [94]. Adding cognitive tasks to the screening tools for fall risk assessment could be one way to increase their sensitivity, but this has been done in a very limited number of studies. The difference between fallers and non-fallers in CoP area and medio-lateral CoP velocity during parallel stance was unchanged when a cognitive task was added, while the difference in CoP antero-posterior velocity increased (SMD from 0.26 to 0.37). Previous studies have shown that dual task-based tests are very useful in predicting falls in older people with cognitive impairment [95]. Similarly, the capability of the Timed-Up-and-Go test to classify fallers and non-fallers was also shown to increase with the addition of a cognitive task [96]. However, our analyses do not support the addition of a cognitive task to the assessment of CoP parameters during quiet stance or non-instrumented balance tests, though only two studies included cognitive tasks. 

Despite the clear indications that CoP parameters could be very useful for the assessment of risk of falling, it remains unclear which tasks (i.e., stance, with or without vison or cognitive task) should be used, and which CoP-related outcome measure should be of primary interest, especially when it comes to the cut-off values for the prediction of falls. This can be attributed in large part to different protocols of CoP analysis in terms of number of repetitions, duration of repetitions, units of measurement, data processing and equipment. Future research would benefit from establishing a unified protocol to facilitate easier comparisons and consequently the determination of absolute cut-off values. For the time being, we can recommend the researchers and clinicians to focus on primarily CoP area, amplitude and path outcomes, and to select those protocols that were proven to be reliable and sensitive. Typically, previous studies have used the average value of 3 repetitions lasting 30 s, though there are indications in the literature that higher durations might be needed to maximize the reliability [97]. Moreover, we encourage future researchers to report the relative changes from condition to condition (e.g., eyes open vs. eyes closed, with vs. without cognitive task), as these measures may represent an additional insight into somatosensory function underlying postural control. The major advantage of such measures is their independence of the mean sample values and units of measurement. This approach has been used before for the Romberg test. Namely, the ratio between the result in eyes open and eyes closed conditions (also termed the Romberg’s quotient) has been suggested to identify differences between fallers and non-fallers because it measures an individual’s reliance on visual input for postural control [41,98]. However, there seems to be no evidence yet that this quotient is associated with the risk of falling, which opens an opportunity for future research in this view.

A major drawback of the present review is the unbalanced representation of different test and outcome measures across studies. Nevertheless, the single-leg stance test can be recommended for clinicians to use for brief assessment of risk of falling, potentially in combination with non-balance tests, such as the Timed-Up-and-Go test. For a more comprehensive analysis, body sway measures, notably CoP area and CoP amplitude should be added to the battery of tests for assessing fall risk. Future research is clearly needed to determine the usefulness of different body sway outcome measures (different combinations of stance, vision condition, surface and parameter). Another major limitation of the current literature is the diversity of the suggested cut-off values for classifying individual as being at high risk for falls. Since this value probably depends on several factors, such as age, general functional ability and the presence of diseases, it is perhaps the best for practitioners to identify individuals with the lowest level of balance ability among their clients (e.g., by using single-leg stance test or body sway assessment) and prescribe them specific exercises for prevention of falls [5,99], while different exercises [100,101,102,103] might be more appropriate for others based on their physical condition (e.g., primarily targeting sarcopenia or other ageing-related problems).

## Figures and Tables

**Table 1:** Differences between fallers and non-fallers according to different outcomes and balance tests.

Test/Outcome Measure	SMD (95% CI) *	Statistical Significance (*p*)	Number of Studies and Heterogeneity (I^2^)	Number of Participants (F/NF)
Instrumented body sway analysis				
CoP Velocity—Parallel EO	0.12 (−0.04, 0.29)	0.140	12 (I^2^ = 42%)	486/823
CoP Velocity—Parallel EC	0.21 (−0.01, 0.43)	0.060	7 (I^2^ = 48%)	290/448
CoP Velocity—Parallel Foam EO	0.29 (−0.19, 0.78)	0.230	1 (I^2^ = N/A)	19/124
CoP Velocity—Semi-tandem EO	−0.01 (−0.72, 0.69)	0.970	2 (I^2^ = 86%)	121/109
CoP Velocity—Parallel EO + COG	0.05 (−0.25, 0.35)	0.730	2 (I^2^ = 0%)	123/71
CoP Velocity AP—Parallel EO	0.26 (0.06, 0.46)	0.010	9 (I^2^ = 63%)	547/972
CoP Velocity AP—Parallel EC	0.17 (0.05, 0.28)	0.004	7 (I^2^ = 0%)	473/863
CoP Velocity AP—Tandem EO	0.09 (−0.17, 0.34)	0.500	2 (I^2^ = 0%)	7/264
CoP Velocity AP—Single-leg	0.96 (0.62, 1.31)	<0.001	1 (I^2^ = N/A)	50/120
CoP Velocity AP—Parallel EO + COG	0.37 (−0.04, 0.77)	0.070	2 (I^2^ = 48%)	75/297
CoP Velocity ML—Parallel EO	0.24 (0.09, 0.38)	0.002	8 (I^2^ = 0%)	317/591
CoP Velocity ML—Parallel EC	0.30 (0.14, 0.47)	<0.001	6 (I^2^ = 0%)	243/482
CoP Velocity ML—Tandem EO	0.06 (−0.20, 0.31)	0.660	2 (I^2^ = 0%)	79/264
CoP Velocity ML—Single-leg	0.33 (−0.01, 0.66)	0.052	1 (I^2^ = N/A)	50/120
CoP Velocity ML—Parallel EO + COG	0.24 (−0.18, 0.66)	0.260	2 (I^2^ = 0.52%)	75/297
CoP Area—Parallel EO	0.60 (0.20, 1.00)	0.003	11 (I^2^ = 90%)	591/2649
CoP Area—Parallel EC	0.30 (0.07, 0.54)	0.010	7 (I^2^ = 65%)	525/2443
CoP Area—Parallel Foam EO	0.57 (0.21, 0.93)	0.002	2 (I^2^ = 0%)	37/191
CoP Area—Single-leg EO	0.66 (0.04, 1.28)	0.040	1 (I^2^ = N/A)	15/35
CoP Area—Single-leg EC	−0.32 (−0.93, 0.29)	0.300	1 (I^2^ = N/A)	15/35
CoP Area—Parallel EO + COG	0.56 (0.08, 1.04)	0.020	1 (I^2^ = N/A)	34/36
CoP Amplitude AP—Parallel EO	0.24 (0.03, 0.45)	0.020	7 (I^2^ = 51%)	341/729
CoP Amplitude AP—Parallel EC	0.15 (−0.01, 0.31)	0.070	6 (I^2^ = 13%)	285/675
CoP Amplitude AP—Parallel Foam EO	0.71 (−0.21, 1.63)	0.130	2 (I^2^ = 85%)	37/191
CoP Amplitude AP—Semi-tandem EO	0.25 (−0.12, 0.63)	0.190	1 (I^2^ = N/A)	56/54
CoP Amplitude AP—Single-leg EO	0.80 (0.06, 1.55)	0.040	1 (I^2^ = N/A)	15/15
CoP Amplitude ML—Parallel EO	0.35 (0.12, 0.58)	0.003	8 (I^2^ = 65%)	430/764
CoP Amplitude ML—Parallel EC	0.38 (0.12, 0.64)	0.004	6 (I^2^ = 62%)	285/675
CoP Amplitude ML—Parallel Foam EO	0.62 (0.27, 0.98)	<0.001	2 (I^2^ = 0%)	37/191
CoP Amplitude ML—Semi-tandem EO	0.21 (−0.16, 0.59)	0.260	1 (I^2^ = N/A)	56/54
CoP Amplitude ML—Single-leg EO	1.31 (0.51, 2.10)	0.002	1 (I^2^ = N/A)	15/15
CoP Path—Parallel EO	0.34 (0.12, 0.56)	0.003	9 (I^2^ = 71%)	597/2284
CoP Path—Parallel EC	0.26 (0.15, 0.37)	<0.001	3 (I^2^ = 0%)	376/2079
CoP Path—Parallel Foam EO	0.51 (0.07, 0.95)	0.020	2 (I^2^ =20%)	39/144
CoP Path—Semi-tandem EO	−0.03 (−0.70, 0.64)	0.930	2 (I^2^ =85%)	121/109
CoP Path AP—Parallel EO	0.63 (−0.01, 1.28)	0.060	3 (I^2^ = 74%)	95/96
CoP Path AP—Parallel EC	1.00 (0.14, 1.85)	0.020	1 (I^2^ = N/A)	12/12
CoP Path ML—Parallel EO	0.58 (0.06, 1.10)	0.030	3 (I^2^ = 61%)	95/96
CoP Path ML—Parallel EC	3.14 (1.88, 4.39)	<0.001	1 (I^2^ = N/A)	12/12
CoP Frequency AP—Parallel EO	0.14 (−0.32, 0.60)	0.550	3 (I^2^ = 70%)	122/189
CoP Frequency AP—Parallel EC	0.05 (−0.40, 0.51)	0.820	3 (I^2^ = 71%)	142/159
CoP Frequency AP—Semi-tandem EO	−0.50 (−0.87, −0.14)	0.007	1 (I^2^ = N/A)	65/55
CoP Frequency ML—Parallel EO	0.12 (−0.29, 0.53)	0.570	3 (I^2^ = 63%)	122/189
CoP Frequency ML—Parallel EC	−0.09 (−0.64, 0.46)	0.750	3 (I^2^ =79%)	142/159
CoP Frequency ML—Semi-tandem EO	−0.42 (−0.79, −0.06)	0.020	1 (I^2^ = N/A)	65/55
Computerized board test—EO, Stable	0.09 (−0.05, 0.24)	0.220	1 (I^2^ = N/A)	232/746
Computerized board test—EC, Stable	0.10 (−0.04, 0.25)	0.160	1 (I^2^ = N/A)	232/746
Computerized board test—EO, Unstable	0.07 (−0.08, 0.22)	0.340	1 (I^2^ = N/A)	232/746
Non-instrumented balance tests				
Functional Reach Test	−0.33 (−0.62, −0.04)	0.030	17 (I^2^ = 91%)	824/2593
Single-leg ST—EO	−0.56 (−0.95, −0.18)	0.004	14 (I^2^ = 94%)	807/2259
Singe-leg ST—EC	−0.03 (−0.38, 0.32)	0.870	4 (I^2^ = 47%)	123/186
Tandem ST—EO	−0.44 (−0.88, 0.00)	0.050	1 (I^2^ = N/A)	26/84
Tandem ST—EC	−0.16 (−0.60, 0.28)	0.470	1 (I^2^ = N/A)	26/84
Romberg Parallel—EO	−0.25 (−0.74, 0.24)	0.320	1 (I^2^ = N/A)	32/32
Romberg Parallel—EC	−0.46 (−0.95, 0.04)	0.070	1 (I^2^ = N/A)	32/32
Romberg Parallel Foam—EO	−0.89 (−1.40, −0.37)	<0.001	1 (I^2^ = N/A)	32/32
Romberg Parallel Foam—EC	−0.52 (−1.02, −0.02)	0.040	1 (I^2^ = N/A)	32/32
Romberg Parallel Foam with Visual Disturbance	−0.57 (−1.07, −0.07)	0.020	1 (I^2^ = N/A)	32/32
Romberg (sum of 4 conditions)	−0.17 (−0.31, −0.02)	0.030	1 (I^2^ = N/A)	232/746

SMD—Standardized mean difference; *—positive SMD indicates higher value in fallers; CoP—center of pressure; F—fallers; NF—non-fallers; EO—eyes open; EC—eyes closed; AP—antero-posterior; ML—medio-lateral; COG—additional cognitive task; ST—stance test; Among the non-instrumented balance tests, the Romberg test in parallel stance with eyes open had the highest SMD (−0.89 (−1.40, −0.37)); however, this was based only on one study. Across the tests that were more frequently used, the single leg stance test with eyes open had higher SMD (0.56 (0.95, −0.18) compared to the functional reach test (SMD = −0.33 (−0.62, −0.04).

**Table 2:** Risk for occurrence of falls, associated with the results of balance tests.

Test / Outcome Measure *	Type of Risk Measure	Risk Measure with 95% CI	Cut-Off/Comparison and Study Reference
CoP Velocity AP—Parallel EO	Odds ratio	1.98 (1.16–3.40)	Lowest to highest quartile [27]
CoP Area—Parallel EC	Odds ratio	1.03 (1.01–1.05)	Per 1 cm^2^ increase [43]
CoP Amplitude AP—Parallel EO	Odds ratio	1.30 (0.60–3.00)	>4.8 mm [77]
CoP Amplitude AP—Parallel EC	Odds ratio	1.50 (0.70–3.60)	>6.7 mm [77]
CoP Amplitude ML—Parallel EO	Odds ratio	2.9 (1.3–6.8)	>4.6 mm [77]
CoP Amplitude ML—Parallel EC	Odds ratio	2.3 (1.00–5.4)	>6.8 mm [77]
CoP Path—Parallel EO	Odds ratio	1.90 (1.27–2.84)	Being in lowest quintile [42]
	Odds ratio	2.00 (0.9–4.69	>67.0 cm (30 s trial) [77]
CoP Path—Parallel EC	Odds ratio	1.65 (1.07–2.55)	Being in lowest quintile [42]
	Odds ratio	1.00 (0.40–2.30)	>113 cm (30 s trial) [77]
Functional Reach Test	Relative risk	1.10 (0.71–1.72)	<18 cm [33]
	Odds ratio	5.28 (0.84–33.2)	<18.5 cm [78]
	Odds ratio	8.67 (2.26–33.29)	<25 cm [55]
	Incidence density ratio	1.12 (0.98–1.28)	Per quintile [34]
	Rate ratio	0.60 (0.50–0.90)	Being in lowest quartile [37]
Single-leg ST—EO	Relative risk	1.62 (1.03–2.56)	<3 s [33]
	Odds ratio	8.54 (4.86–14.99)	<12.7 s [55]
	Odds ratio	15.22 (1.72–133.95)	<1.02 s [78]
	Odds ratio	0.38 (0.17–0.84)	Per 1 standard deviation [86]
Tandem ST—EO	Odds ratio	2.33 (1.34–4.04)	Able/unable [32]
Romberg Parallel—EO	Odds ratio	7.53 (4.58–12.38)	<20 s [55]
Romberg (sum of 4 conditions)	Odds ratio	2.00 (1.21–3.04)	<5 s [22]

* each row represents a different study; CoP—center of pressure; F—fallers; NF—non-fallers; EO—eyes open; EC—eyes closed; AP—antero-posterior; ML—medio-lateral; ST—stance test.

**Table 3:** Sensitivity and specificity of balance test for prediction of falls and suggested cut-off values in different studies.

Table *	Suggested Cut-Off Points/Reference to the Study	Sensitivity	Specificity
CoP Velocity AP—Single-leg	>2.9 cm/s [69]	0.78	0.54
CoP Velocity ML—Single-leg	>3.4 cm/s [69]	0.70	0.58
Functional Reach Test	<4 cm [40]	0.70	0.43
	<18.0 cm [33]	0.47	0.59
	<18.5 cm [78]	0.75	0.67
Single-leg ST—EO	<1.02 s [78]	0.67	0.89
	<3.0 s [33]	0.51	0.61
	<8.0 s [40]	0.67	0.48

* each row represents a different study; CoP—center of pressure; AP—antero-posterior; ML—medio-lateral; EO—eyes open.
